# Supplementary figures and images for: Identification and validation of an endoplasmic-reticulum-stress-related gene signature as an effective diagnostic marker of endometriosis
Source: PeerJ. 2024 Mar 25;12:e17070. doi: 10.7717/peerj.17070 (PMC10977089; doi:10.7717/peerj.17070)

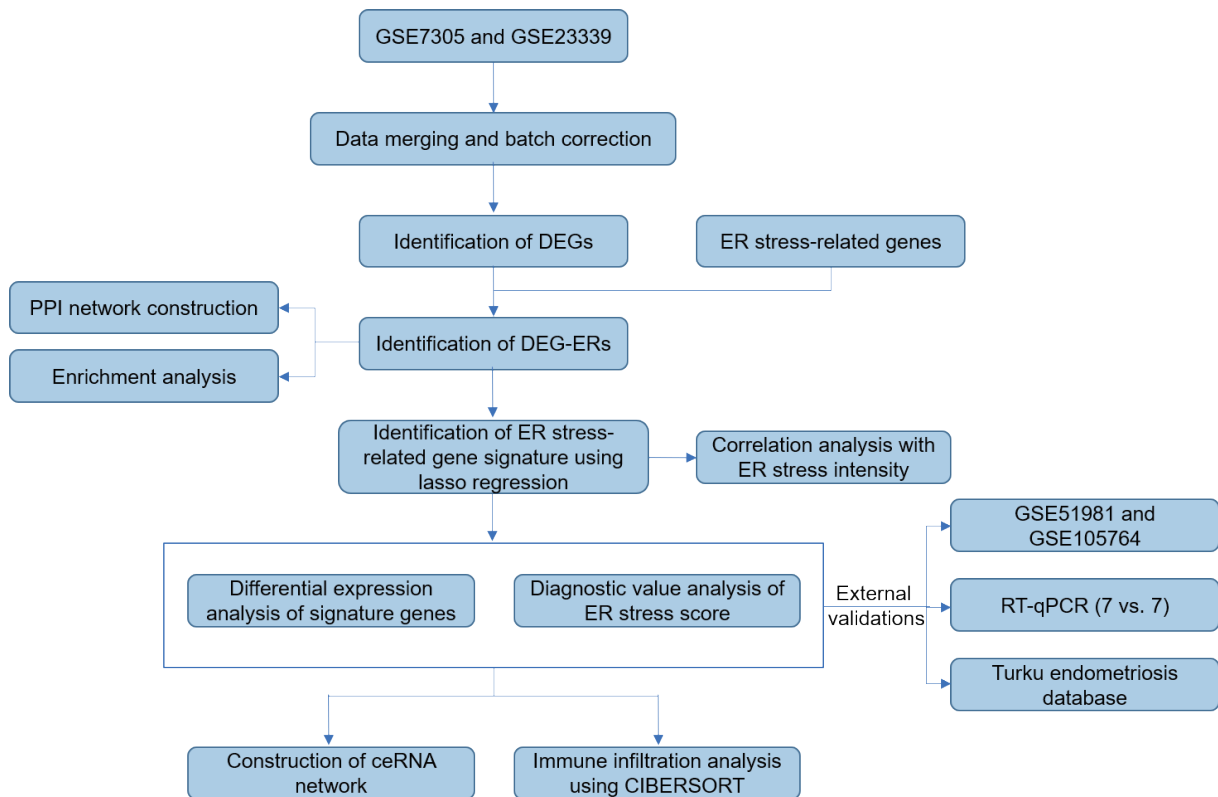

Supplement: Supplemental Information 1 — DEGs, differentially expressed genes; DEG-ERs, differentially expressed ER stress-related genes; PPI, protein-protein interaction; ceRNA, competing endogenous RNA [file peerj-12-17070-s001.pdf]

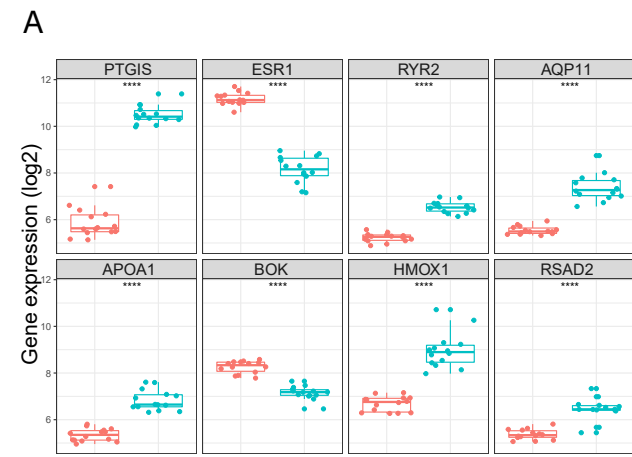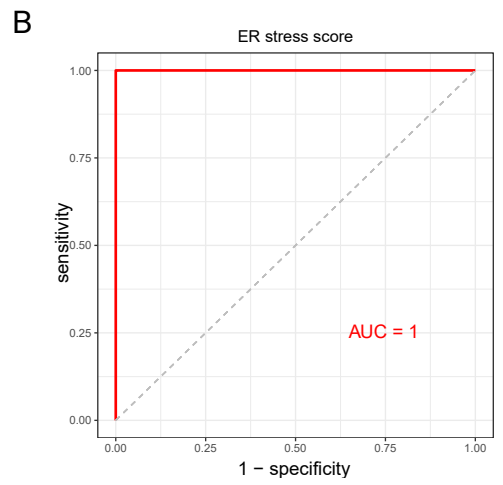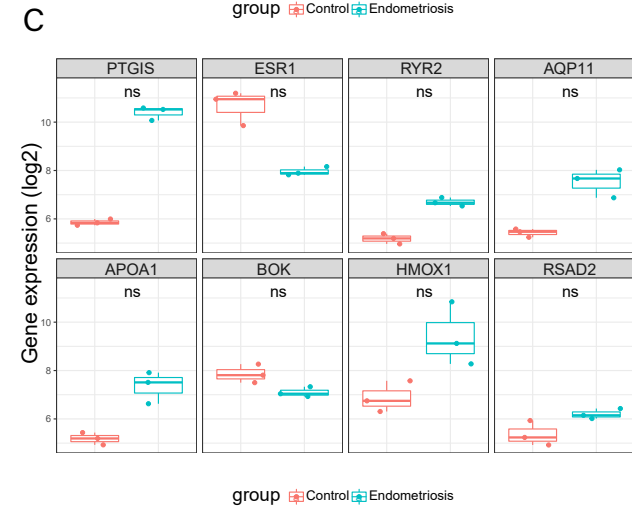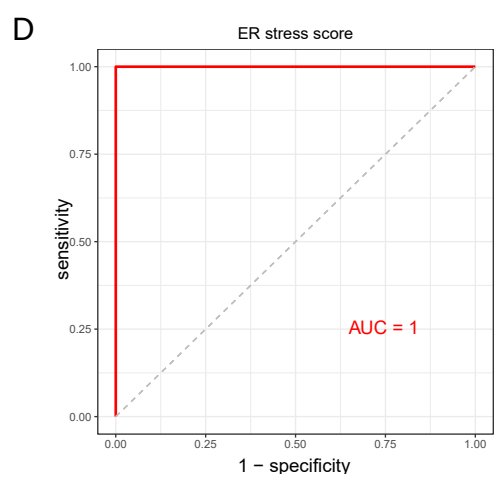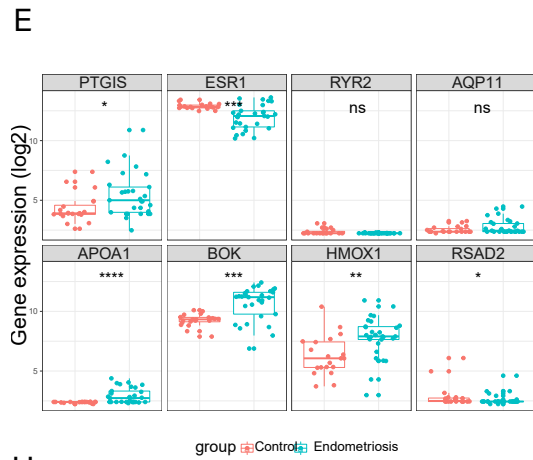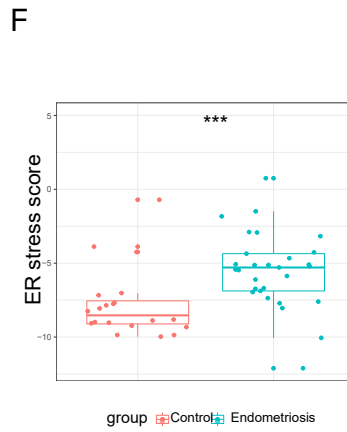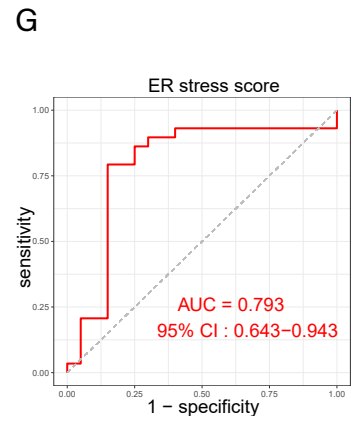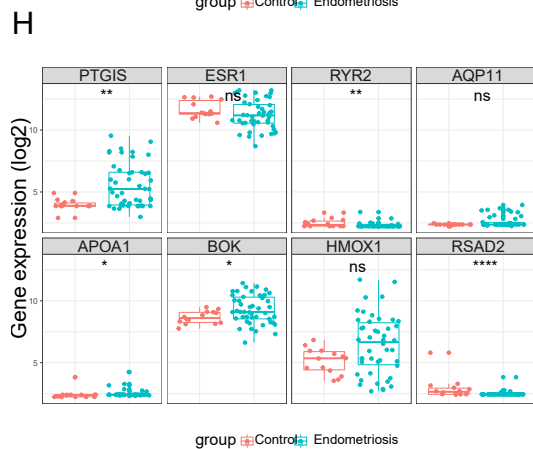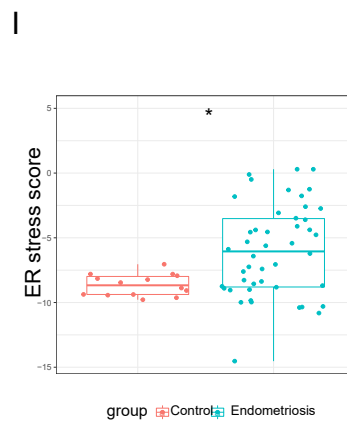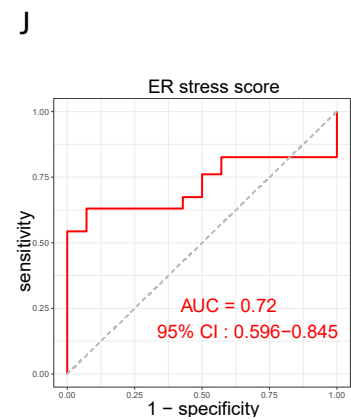

Supplement: Supplemental Information 2 — (A, C) The expression levels of eight signature genes in samples of the training cohort in the proliferative stage (A) and secretory stage (C), respectively. (B, D) ROC analysis for calculated ER stress score in samples of the training cohort in the proliferative stage (B) and secretory stage (D), respectively. (E, H) The expression levels of eight signature genes in samples of the GSE51981 cohort in the proliferative stage (E) and secretory stage (H), respectively. (F, I) The relative ER stress scores of endometriosis samples and normal controls of the GSE51981 cohort in the proliferative stage (B) and secretory stage (E), respectively. (G, J) ROC analysis for calculated ER stress score in samples of the GSE51981 cohort in the proliferative stage (G) and secretory stage (J), respectively. ns, not significant, *p < 0.05, **p < 0.01, ***p < 0.001; ROC, receiver operator characteristic; AUC, area under the curve. [file peerj-12-17070-s002.pdf]

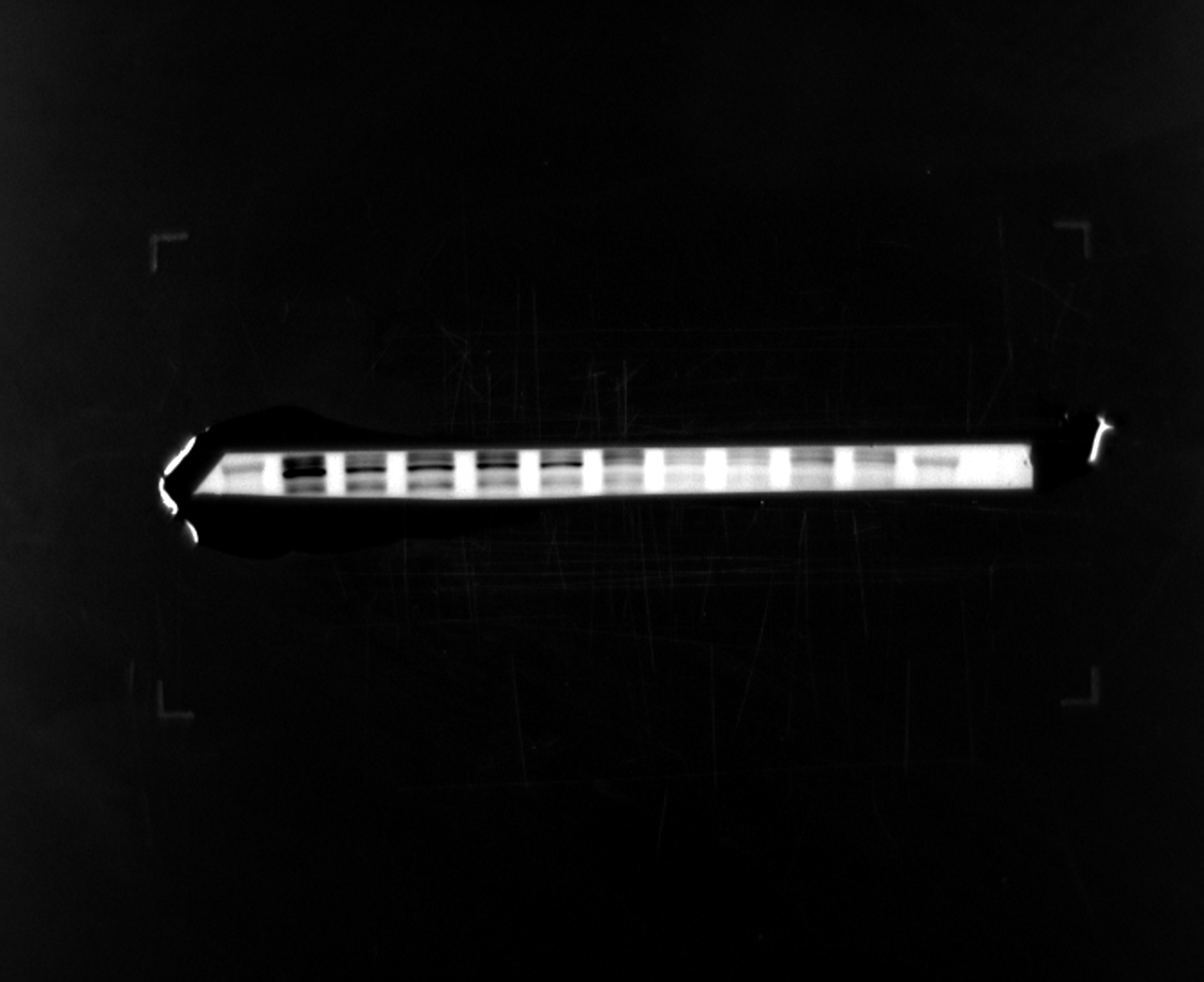

Supplement: Supplemental Information 7 [file peerj-12-17070-s007.zip › ATF6-Merge.Tif]

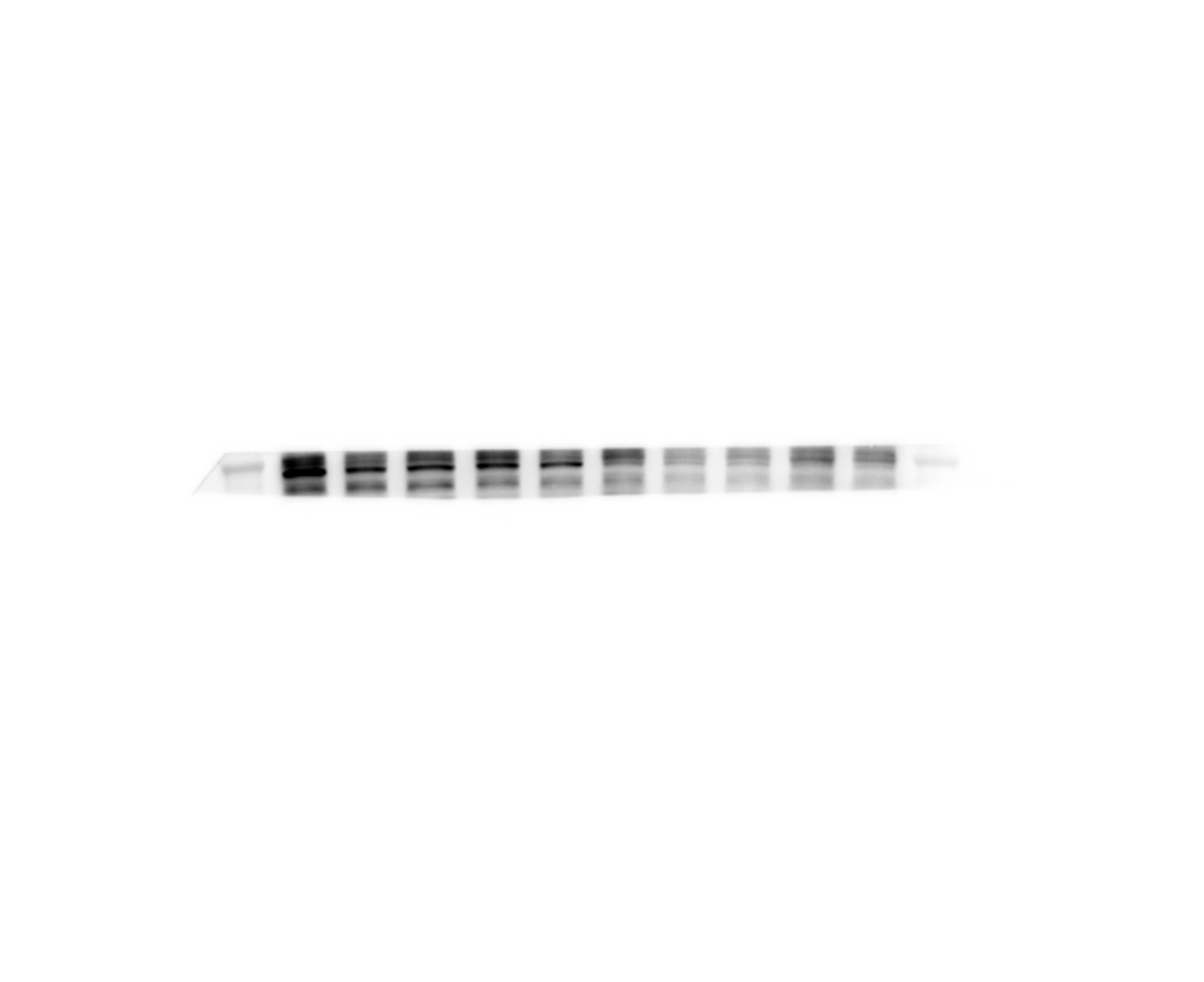

Supplement: Supplemental Information 7 [file peerj-12-17070-s007.zip › ATF6.Tif]

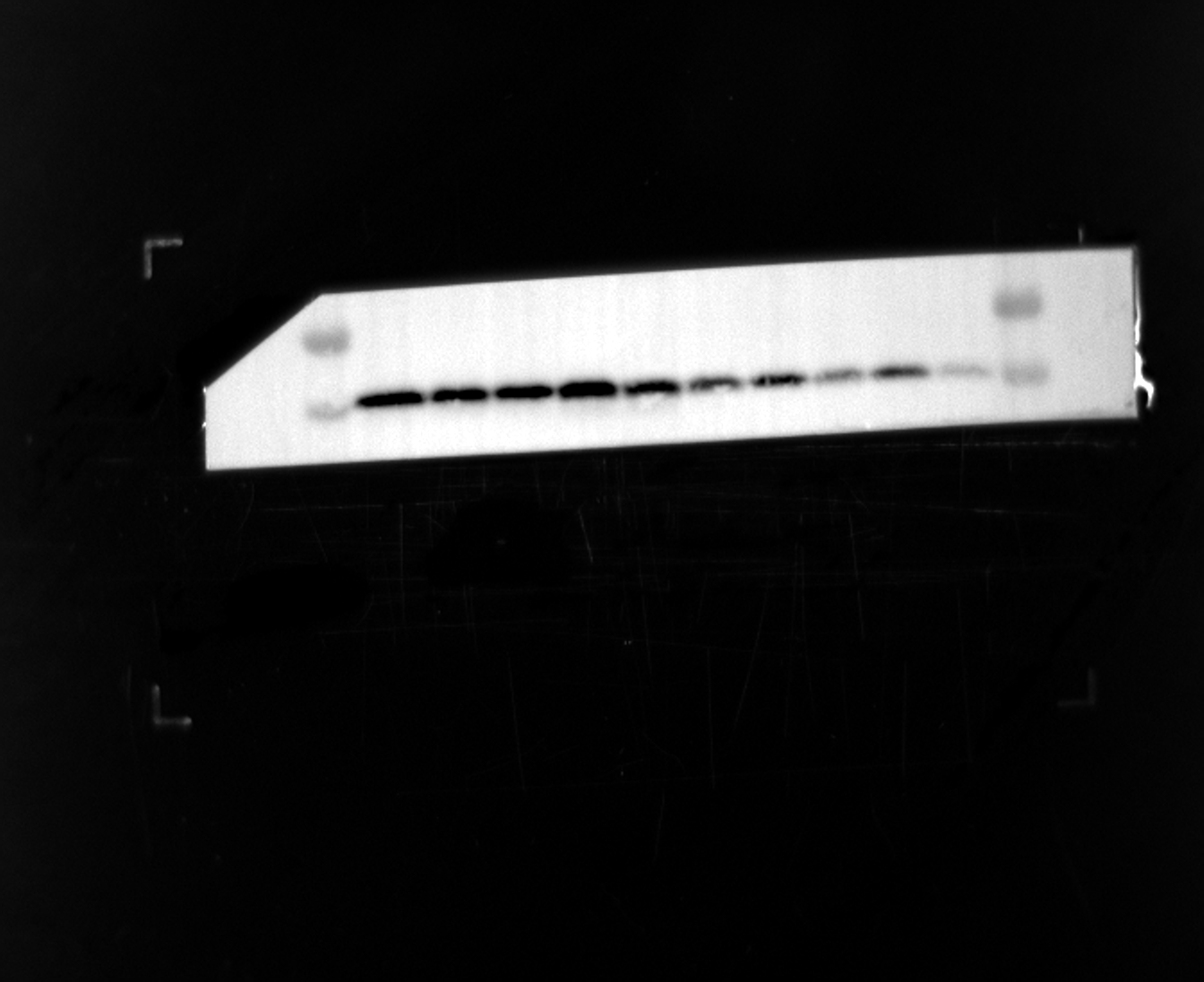

Supplement: Supplemental Information 7 [file peerj-12-17070-s007.zip › BOK-Merge.Tif]

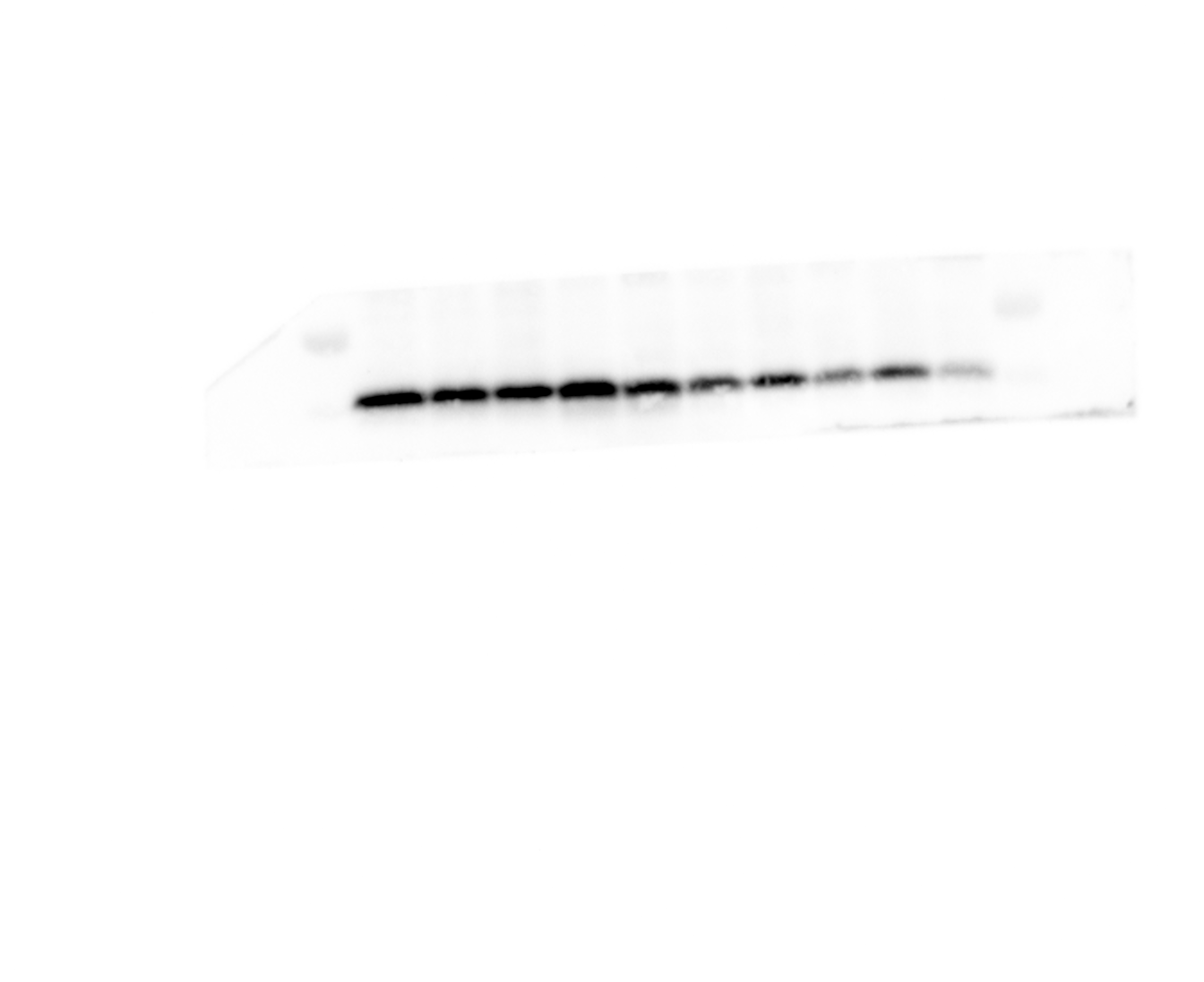

Supplement: Supplemental Information 7 [file peerj-12-17070-s007.zip › BOK.Tif]

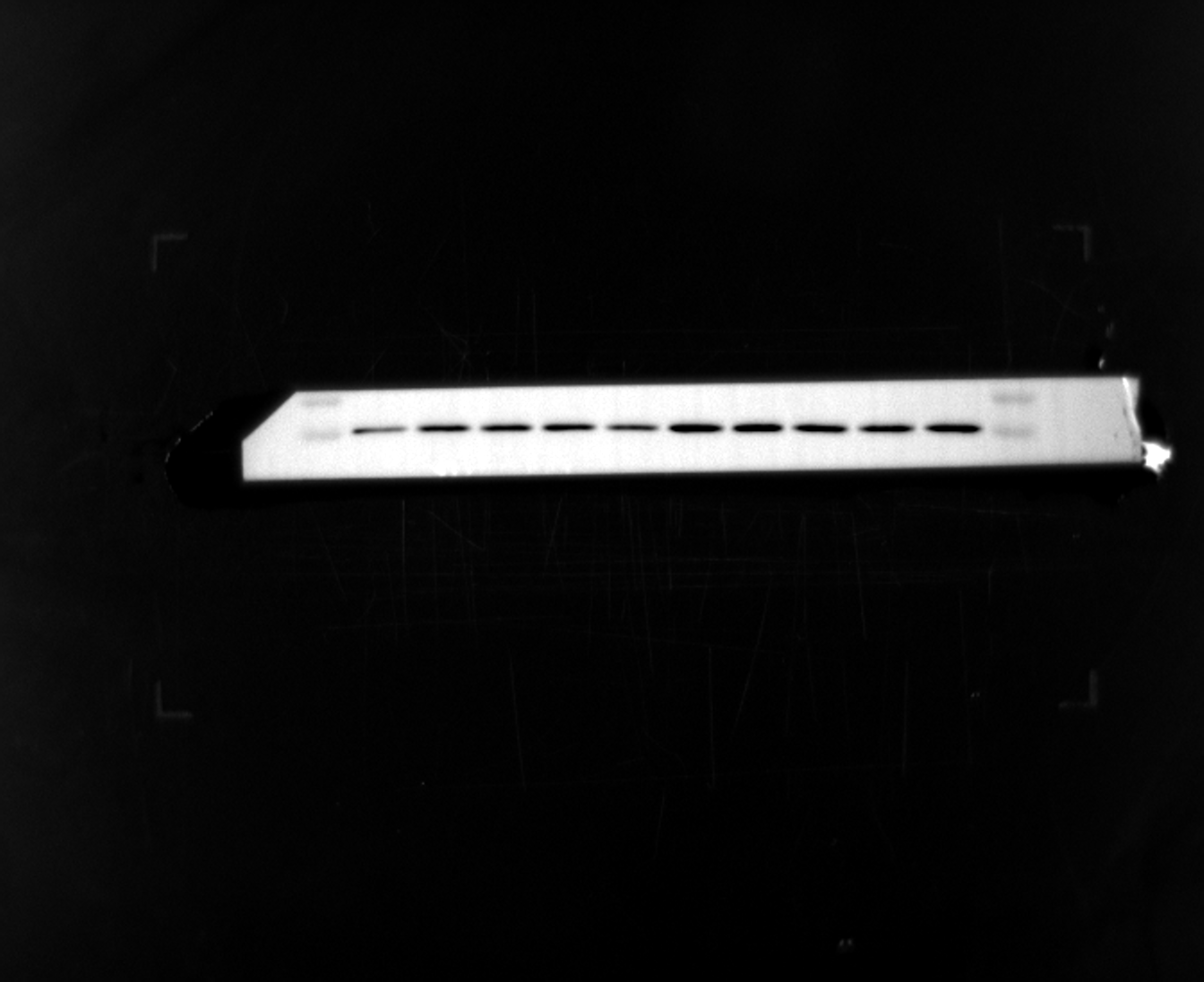

Supplement: Supplemental Information 7 [file peerj-12-17070-s007.zip › GAPDH Merge.Tif]

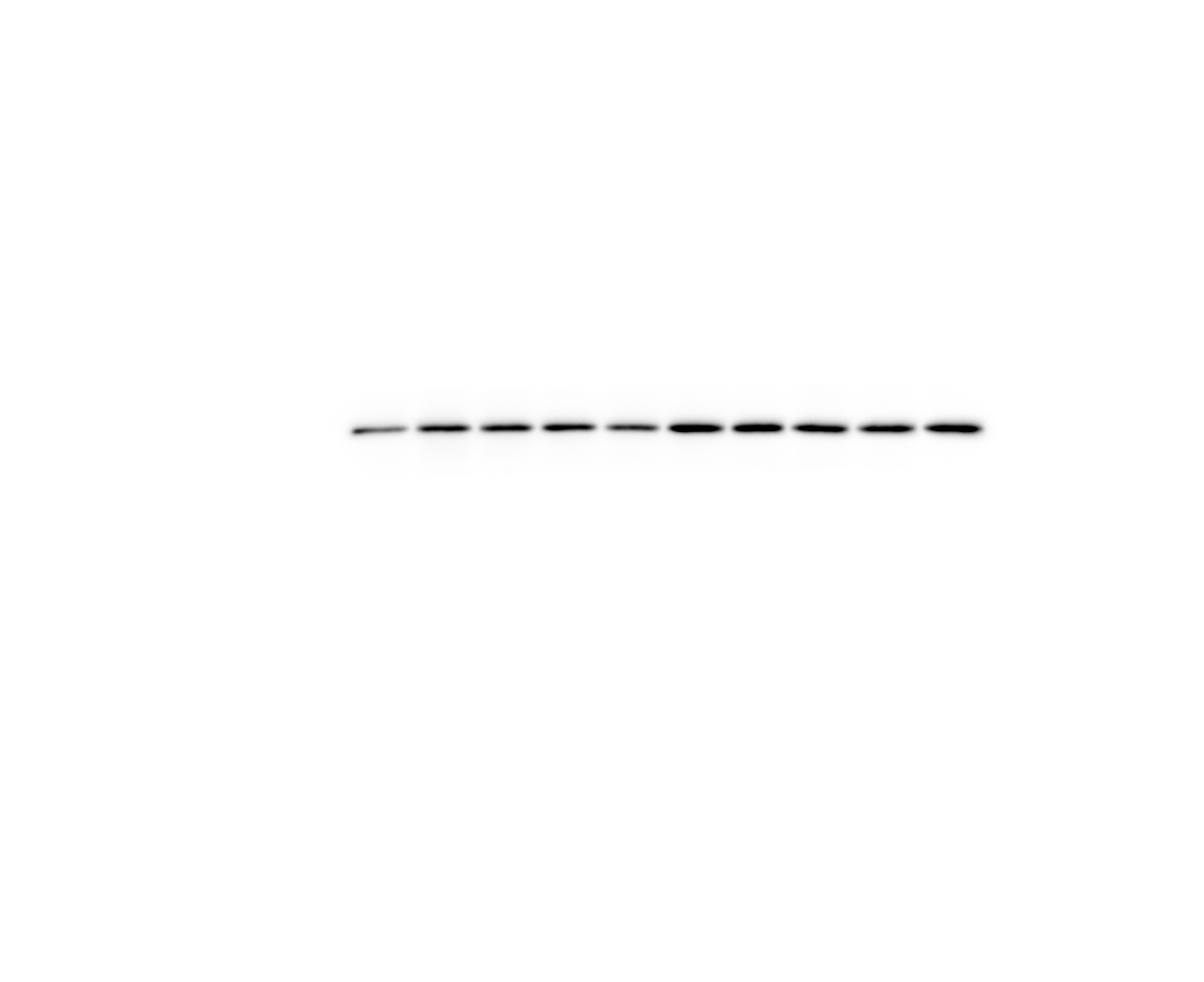

Supplement: Supplemental Information 7 [file peerj-12-17070-s007.zip › GAPDH.Tif]

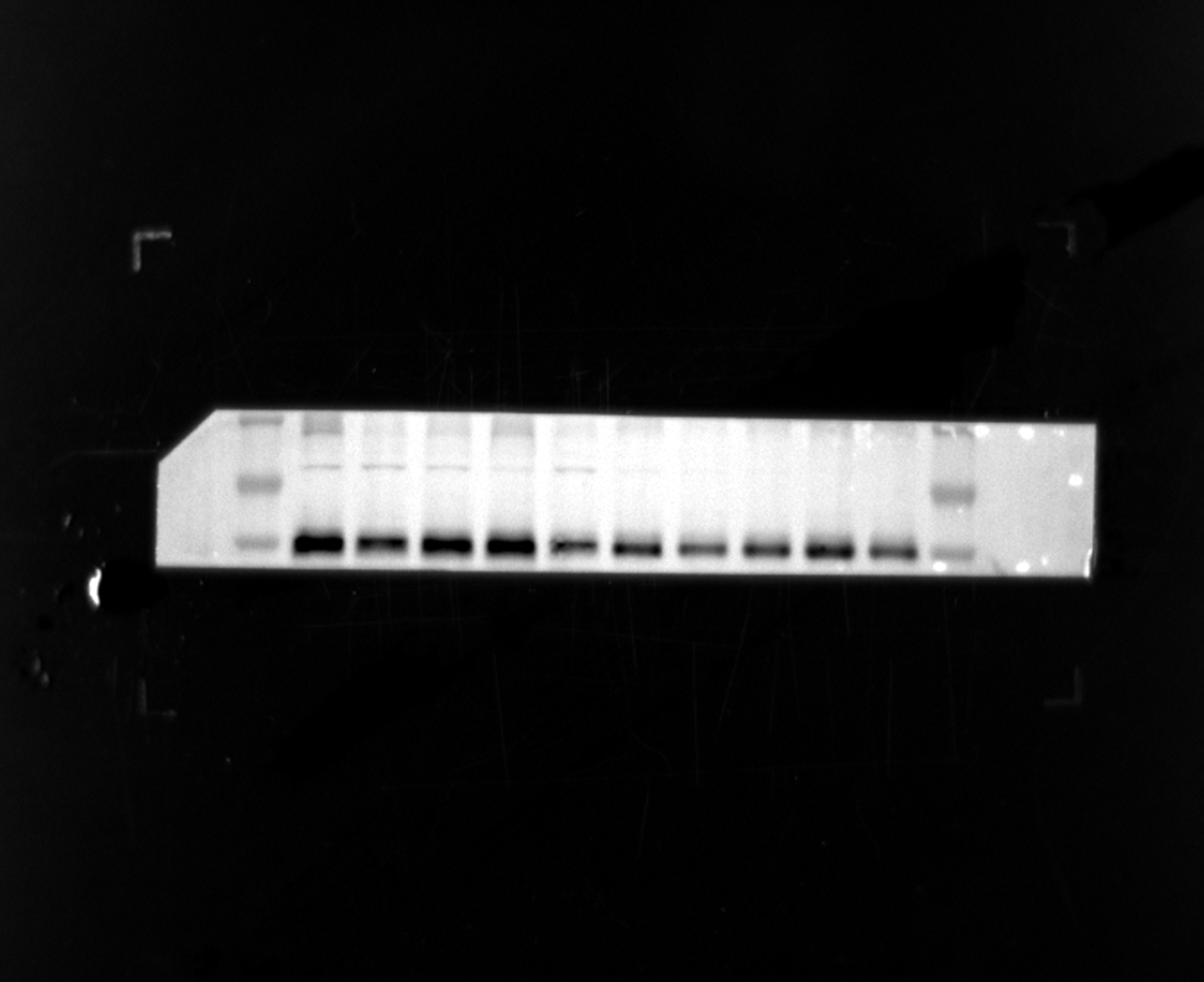

Supplement: Supplemental Information 7 [file peerj-12-17070-s007.zip › GRP78-Merge.Tif]

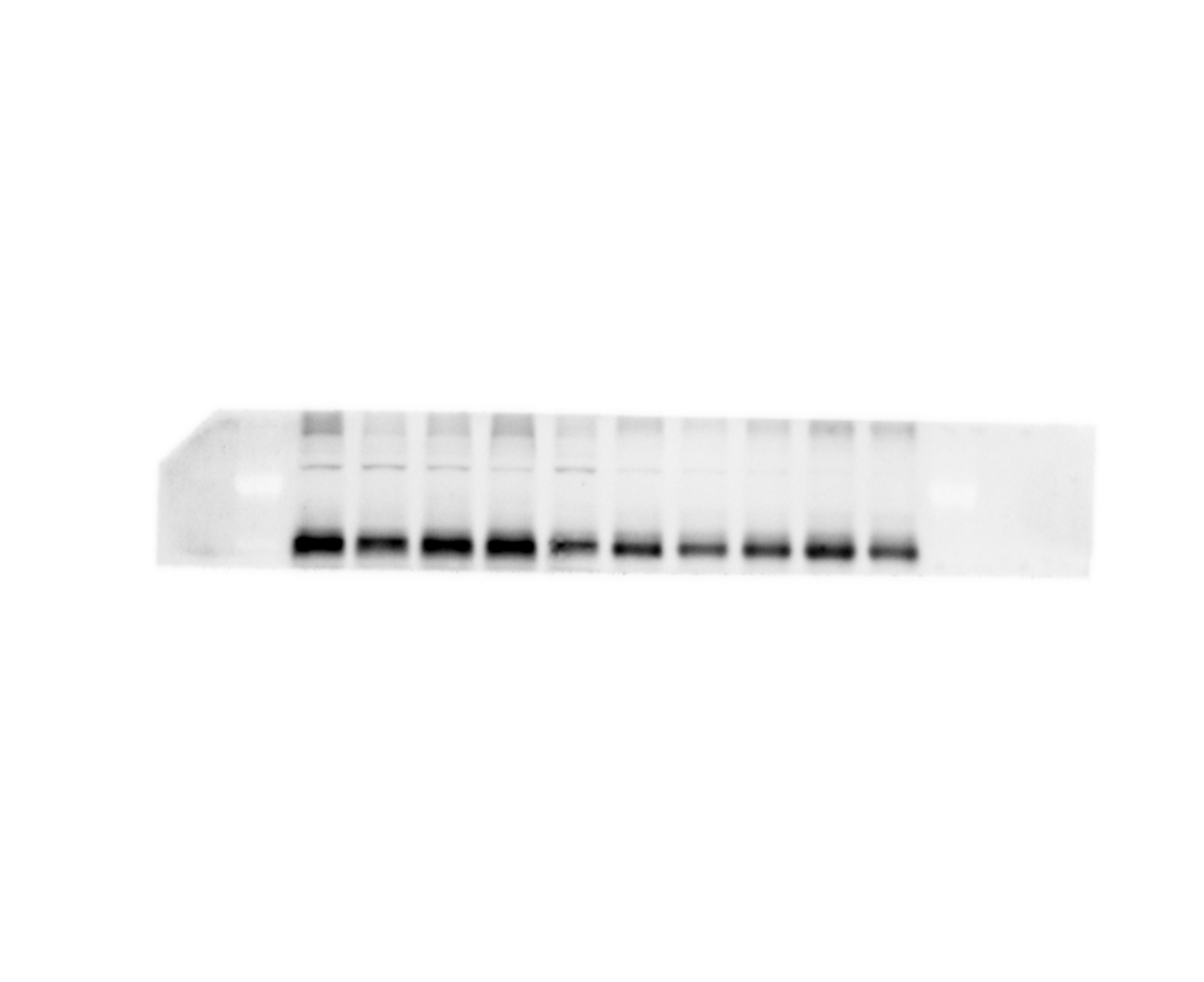

Supplement: Supplemental Information 7 [file peerj-12-17070-s007.zip › GRP78.Tif]

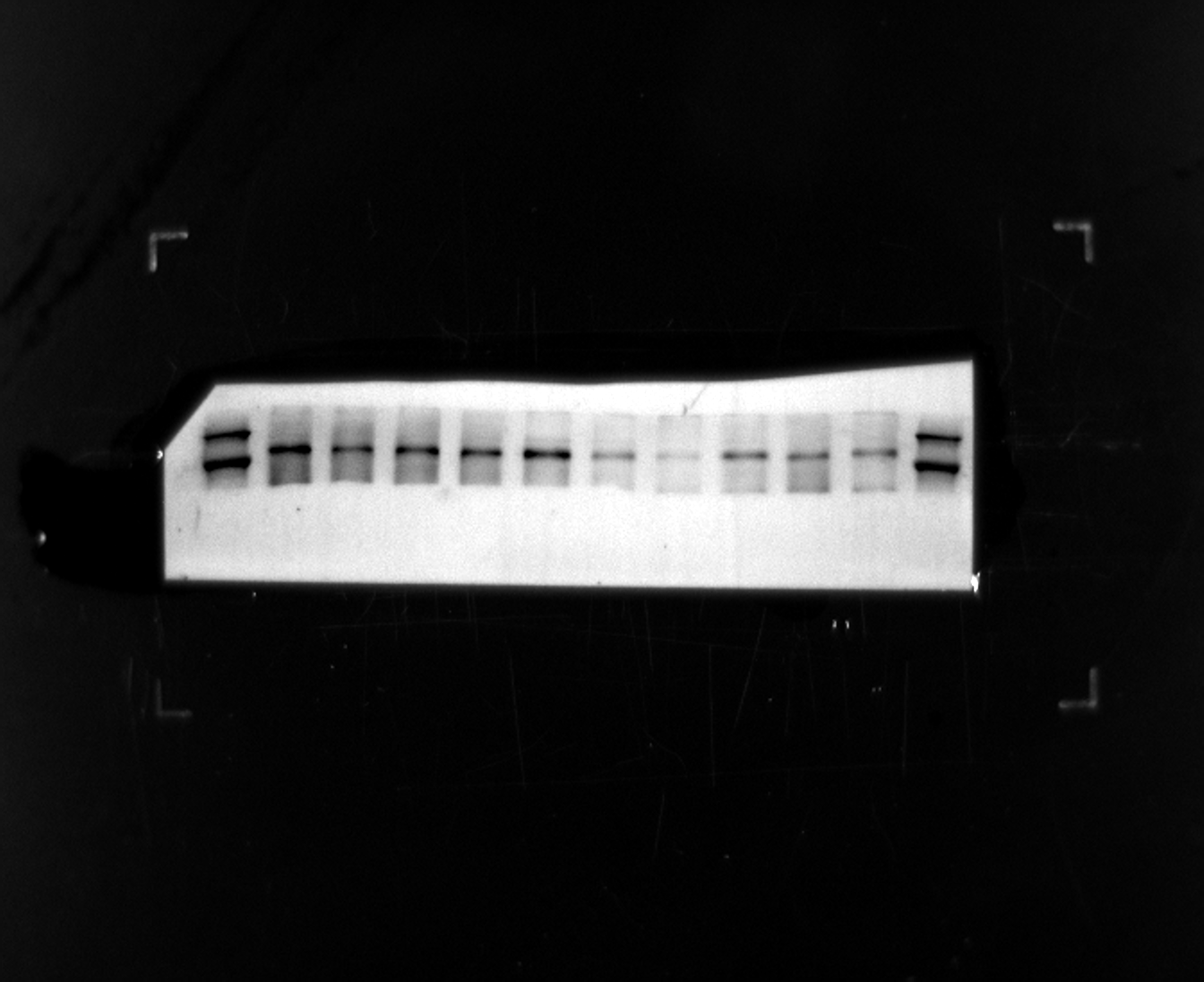

Supplement: Supplemental Information 7 [file peerj-12-17070-s007.zip › p-PERK-Merge.Tif]

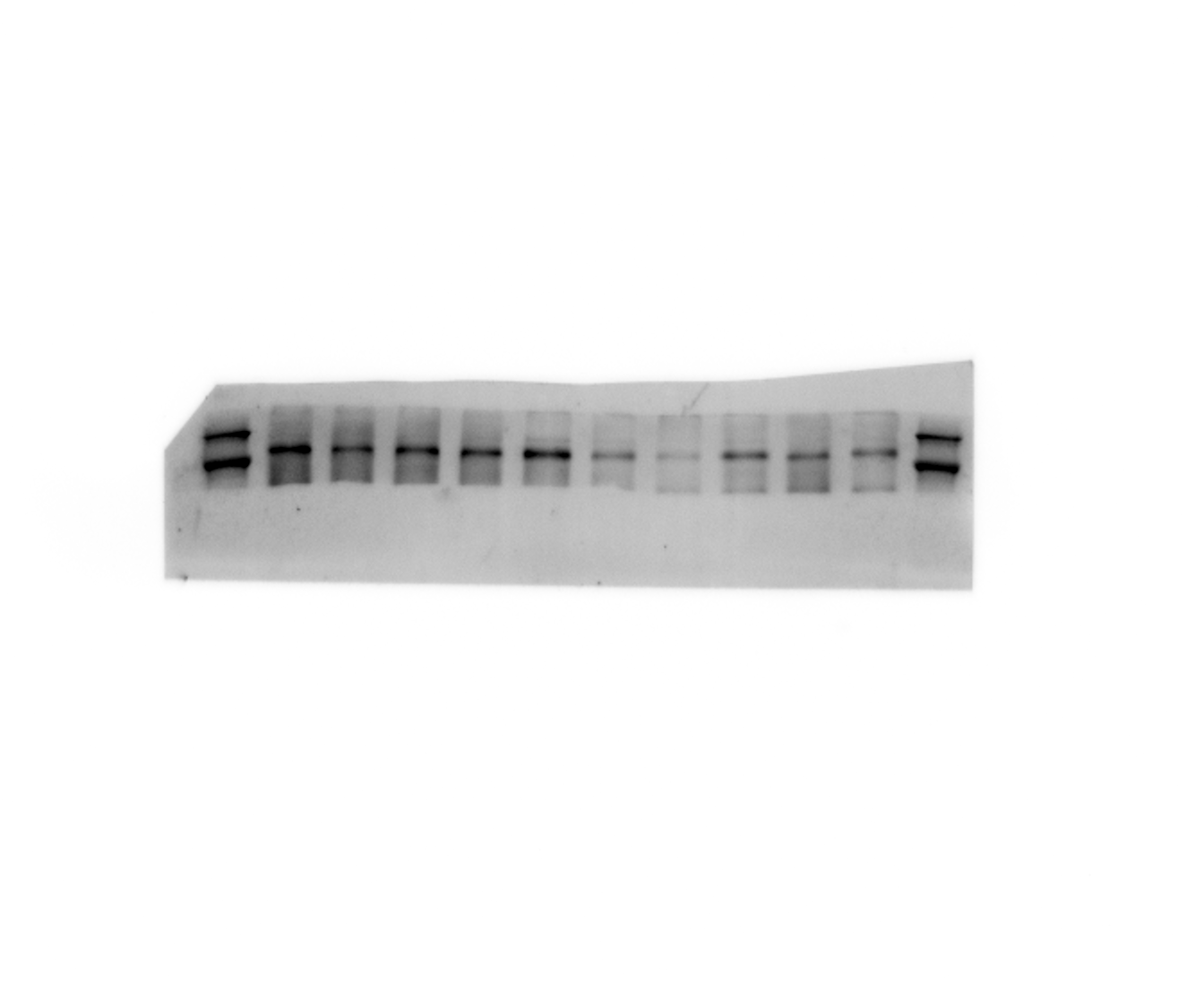

Supplement: Supplemental Information 7 [file peerj-12-17070-s007.zip › p-PERK.Tif]

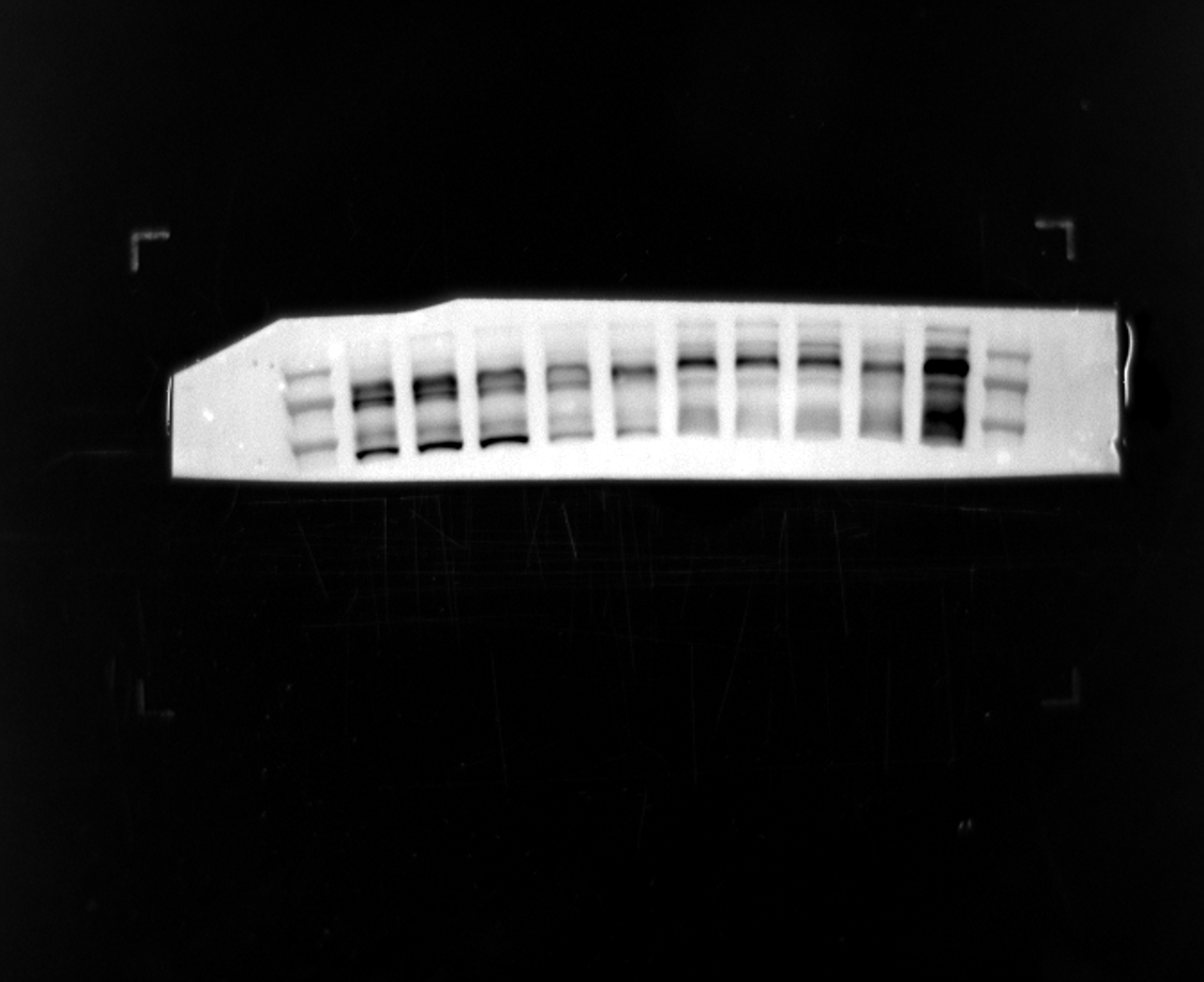

Supplement: Supplemental Information 7 [file peerj-12-17070-s007.zip › perk-Merge.Tif]

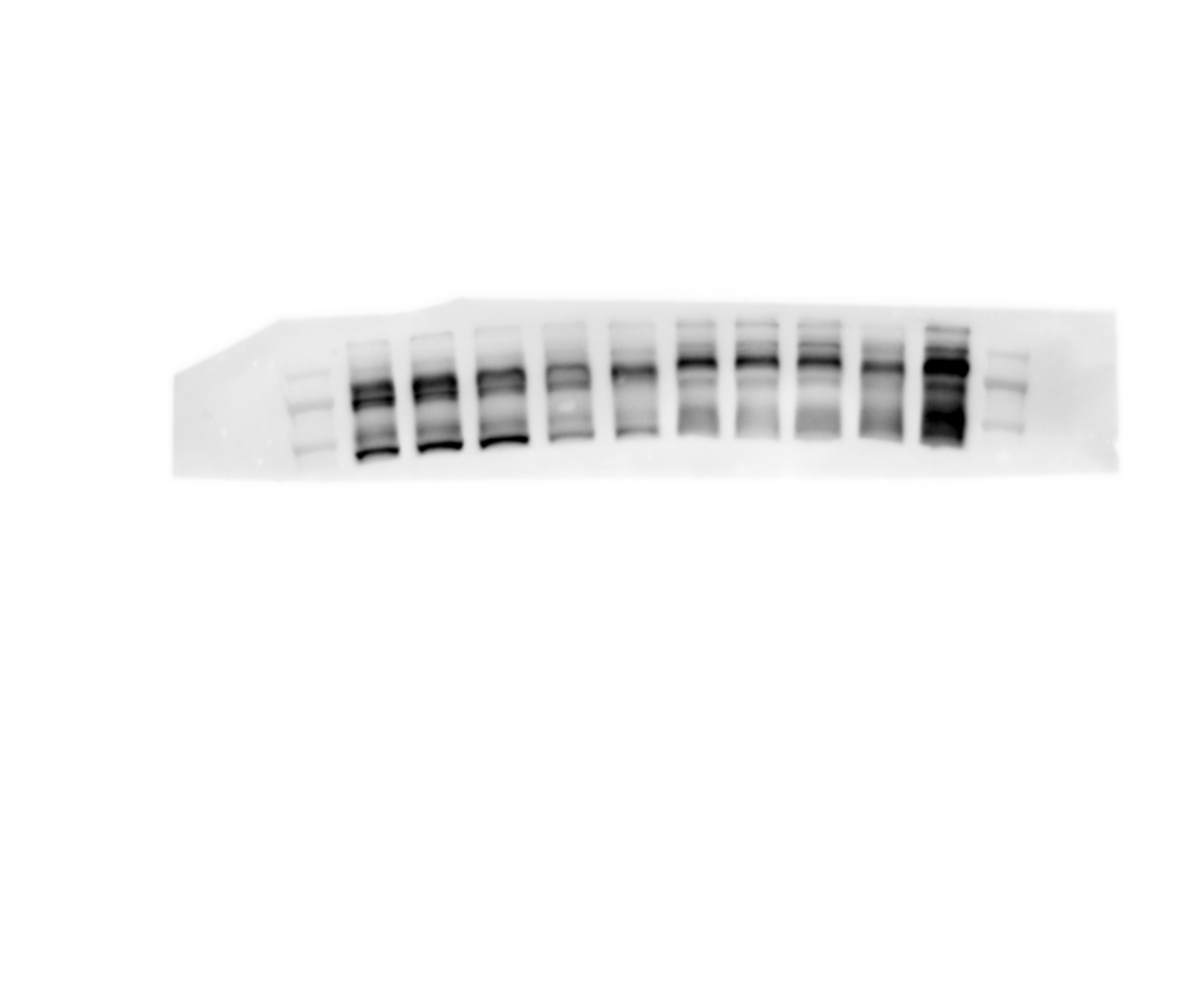

Supplement: Supplemental Information 7 [file peerj-12-17070-s007.zip › perk.Tif]
