# Supplementary material for: Identification and validation of an endoplasmic-reticulum-stress-related gene signature as an effective diagnostic marker of endometriosis
Source: PeerJ. 2024 Mar 25;12:e17070. doi: 10.7717/peerj.17070 (PMC10977089; doi:10.7717/peerj.17070)
Supplement: Supplemental Information 3 — (A-G) The relative differential mRNA levels of PTGIS, ESR1, RYR2, AQP11, APOA1, BOK, HMOX1, RSAD2 among different endometriosis subtypes and normal controls, respectively. [file peerj-12-17070-s003.pdf]

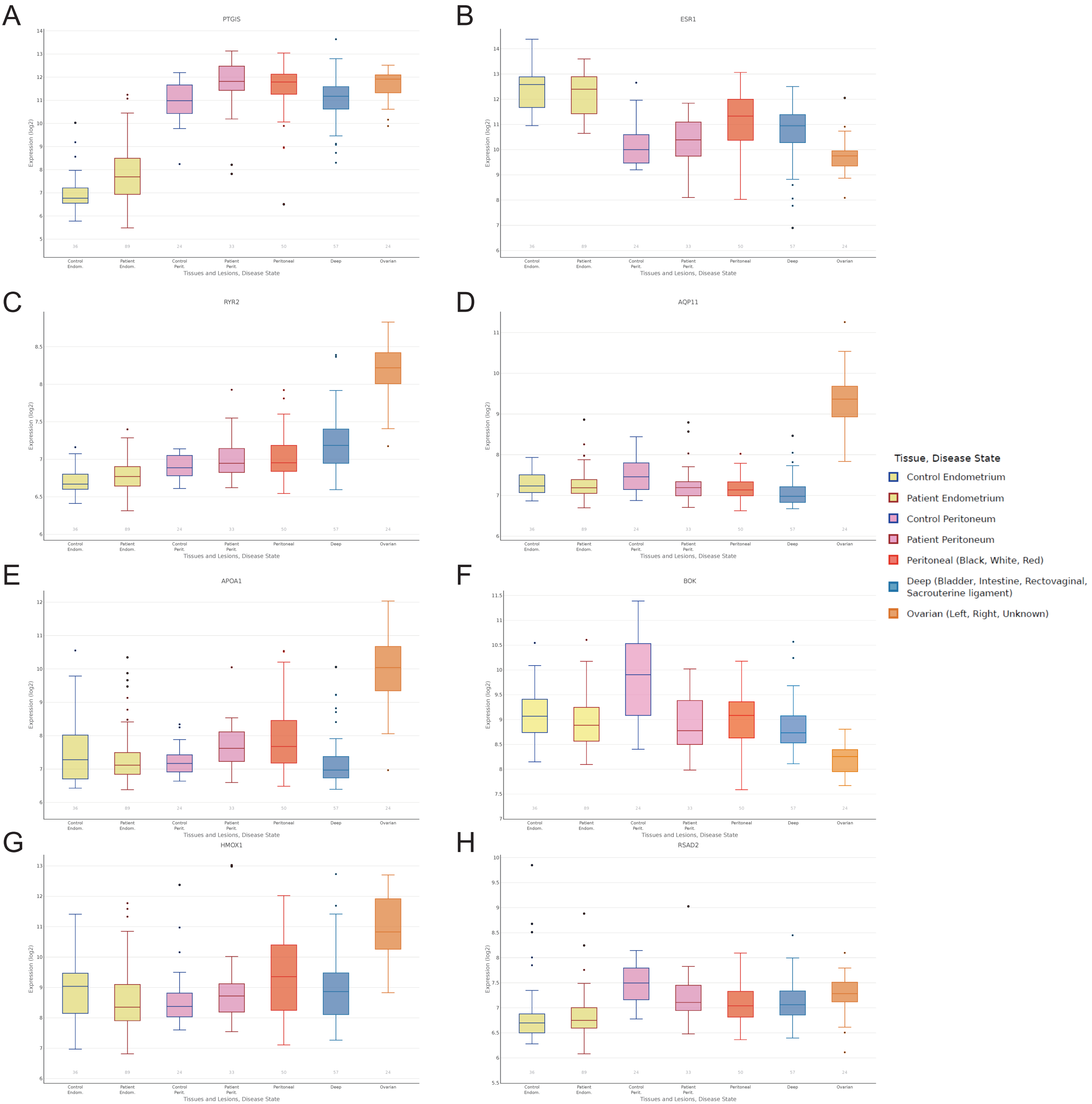

A
PTGIS
B
ESR1
C
RYR2
D
AQP11
E
APOA1
F
BOK
G
HMOX1
H
RSAD2
Expression (log2)
36
89
24
33
50
57
24
Control Endom.
Patient Endom.
Control Perit.
Patient Perit.
Peritoneal
Deep
Ovarian
Tissues and Lesions, Disease State
Tissue, Disease State
Control Endometrium
Patient Endometrium
Control Peritoneum
Patient Peritoneum
Peritoneal (Black, White, Red)
Deep (Bladder, Intestine, Rectovaginal, Sacrouterine ligament)
Ovarian (Left, Right, Unknown)
